# Mast seeding is stronger in taller plants

**DOI:** 10.3389/fpls.2024.1382824

**Published:** 2024-07-09

**Authors:** Haiming Qin, Xianfeng Yi

**Affiliations:** School of Life Sciences, Qufu Normal University, Qufu, China

**Keywords:** mast seeding intensity, plant height, pollination vector, life form, Spermatophyte

## Abstract

**Introduction:**

Two economies of scale, predator satiation and pollination efficiency, have been proposed to explain the evolutionary mechanisms of mast seeding adopted by some long-lived plants. Plant height is strongly selected by pollination vectors and may also provide economies of scale; however, it remains unknown whether there is a close relationship between adult plant height and mast seeding intensity.

**Methods:**

Here, we analyzed mast seeding intensity of 158 plant species to test if adult plant height can select for mast seeding.

**Results:**

We show that mast seeding intensities are higher in taller plant species irrespective of phylogeny, life form, pollination vector, and type of Spermatophytes. We also show that anemophily rather than entomophily selects for taller plant species and higher mast seeding intensities.

**Discussion:**

The linear correlations and evolutionary links between adult plant height and mast seeding intensity provide evidence that mast seeding could have evolved as an adaptation to taller strategy of perennial plant species.

## Introduction

Masting, also called mast seeding or masting behavior, is a widespread reproductive strategy exhibited by some long-lived plant species (Kelly and Sork, 2002; Hacket-Pain and Bogdziewicz, 2021; Koenig, 2021). Masting is defined as the massive, intermittent and synchronous production of seed crops at irregular supra-annual intervals (Herrera et al., 1998; Kelly and Sork, 2002; Koenig and Knops, 2005; Crone et al., 2011), which has been extensively documented in a number of perennial plant species around the world (Kelly, 1994; Kelly and Sork, 2002; Crone and Rapp, 2014; Pearse et al., 2016; LaMontagne et al., 2020). This massive and synchronous variation in seed crops, which is mainly triggered by genetic regulation and/or environmental cues (Burns, 2012; Fleurot et al., 2023), can alter plant-animal interactions and increase the probability of seed survival as well as seedling establishment (Wang and Yi, 2022; Xiao et al., 2022). Ultimately, mast seeding contributes a great importance to population dynamics of perennial plant species (Espelta et al., 2008; Pearse et al., 2021; Zhang et al., 2022).

Due to the crucial role of masting in the regulation of population dynamics of both plant and seed predators (Bogdziewicz et al., 2016; Zeng et al., 2021; Liu et al., 2022; Yu et al., 2023), understanding the ultimate causes of such a widespread reproductive strategy is of great importance to explain the evolution and advantages of mast seeding of plants (Visser et al., 2011; Huang et al., 2021; Seget et al., 2022). It has been suggested that masting can evolve only when costs are minimized followed by the maximized returns (Seget et al., 2022). i.e., greater reproductive efficiency at high reproductive effort (Norton and Kelly, 1988). Three hypotheses based on economies of scale (i.e., increased pollination efficiency and decreased seed predation in mast years) have been proposed on the evolutionary advantages of masting (Kelly et al., 2001; Kelly and Sork, 2002; Linhart et al., 2014; Bogdziewicz et al., 2016; Seget et al., 2022). I: the pollination efficiency hypothesis posits that mast seeding can be selected by increased pollination rates in high flowering years (Kelly et al., 2001; Bogdziewicz et al., 2020). II: the predator satiation hypothesis suggests that satiating predators benefits plants by escaping seed predation through starvation of seed predators between mast years and satiation of predators during mast events (Bogdziewicz et al., 2016, 2018; Greenberg and Zarnoch, 2018; Zhang et al., 2022; Zwolak et al., 2022), and ultimately increases seed dispersal by animals and the chance of seed survival as well as seedling recruitment (Kelly and Sork, 2002; Koenig et al., 2003; Fletcher et al., 2010; Xiao et al., 2013; Zhang et al., 2022). III: the animal dispersal hypothesis, despite less widely accepted than the predator satiation hypothesis, postulates that mast seeding may enhance seed dispersal, increase dispersal distances and finally improve the chances that seeds escape post-dispersal predation (Vander Wall, 2001, 2002; Jansen et al., 2004; Burns, 2012; Hanya and Bernard, 2013; Mendoza et al., 2015), consistent with the predator satiation hypothesis.

Given the importance of economies of scale (EOS) in the evolution of mast seeding, any selective pressures that provide EOS may have the potential to select for mast seeding. Previous work has shown that wind-pollinated species provide economies of scale as opposed to animal-pollinated ones (Kelly and Sork, 2002), indicating that mast seeding intensity has increased selection pressure mainly from pollination vectors. Plant height, one of the most important life-history traits, has been documented to be selected by pollination vectors (Bickel and Freeman, 1993; Donnelly et al., 1998; Zu and Schiestl, 2017; Hernández-Villa et al., 2020). Evidence has been provided for the important role of taller strategy in promoting effective pollen dispersal and seed dispersal either for wind- or animal-pollinated plants (Culley et al., 2002; Friedman and Barrett, 2009; Thomson et al., 2011). Despite of various benefits of taller strategy, the cost of the increased difficulties in transporting water and nutrients between roots and crowns make taller plants more susceptible to various abiotic stresses such as irregular droughts than smaller ones (Kenzo et al., 2006; Cao et al., 2012). In addition, decreased stomatal conductance and assimilation rate of leaves with increasing tree height not only prevent carbon uptake but deplete carbon reserves, leading to drought-induced C-starvation in taller trees (Grote et al., 2016). Moreover, taller plants are usually long-lived and need to put more effort into growth rather than reproduction, which is likely to come at a price of seed production failure. Therefore, comparing with shorter plant species, such a trade-off between benefits and costs is likely to result in selection of mast seeding in taller plants, favoring those with drastic variations in annual seed reproduction. A renowned example has been the giant trees from Dipterocarpoidae exhibiting extremely longer intervals of highly synchronized mast seeding (Wang et al., 2024). Therefore, taller plants are expected to act as a hidden player to make large reproductive efforts more efficient, providing EOS and selection pressure for mast seeding.

Although an increasing number of studies has made great contributions to reveal the underlying mechanisms of masting behavior (Kelly and Sork, 2002; Crone et al., 2009; Turnbull et al., 2011; Kelly et al., 2013; Miyazaki et al., 2014; Pearse et al., 2014, 2016; Fleurot et al., 2023), the potential role of adult plant height in selecting for mast seeding has been largely overlooked. Here, we first collected data of adult plant height and mast seeding intensity of 158 species of spermatophytes with different life forms and pollination vectors. Because comparative studies have identified a moderate phylogenetic signal of masting, i.e., similarity in masting intensity among congeneric plants (Koenig et al., 2016; Fernández-Martínez et al., 2019; Pearse et al., 2020; Dale et al., 2021), we then fitted multivariate phylogenetic generalized linear mixed model (PGLMM) to test whether there is a close relationship between adult plant height and mast seeding intensity at population level rather than individual level, which has been a conventional evaluation method of masting. We specifically predict that mast seeding intensity will co-evolve with adult plant height and that taller plant species will exhibit higher mast seeding intensity. In addition, anemophily adopting taller strategy will show higher mast seeding intensity than entomophily.

## Materials and methods

### Sampling data

The intensity of mast seeding of plant species, expressed as the consecutive disparity index (D), was directly derived from the study of Fernández-Martínez et al. (2019). To calculate the D value of each species, Fernández-Martínez et al. (2019) included two features (temporal variability and lag 1 autocorrelation) of mast seeding into the formula:


$$D=\;\frac{1}{n-1}\sum_{i=1}^{n-1}\left|ln\frac{p_{i+1}+k}{p_{i}+k}\right|$$

Where, D stands for mast seeding of a given species. *pi* and *n* represent the series value at time *i* and series length, respectively. Here, *k* was assigned to a constant 1. Data represented accurate seed or fruit production were included, while perennial species that bears fruit once in its life was excluded. Moreover, in rare cases, records of inflorescence set weakly linked to seed or fruit production were also excluded, e.g., for the umbellate species.

We then compiled data on adult plant height (m), life form (tree vs. non-tree), and type of Spermatophytes (angiosperms vs. gymnosperms) by searching the TRY Plant Trait Database (https://www.try-db.org/TryWeb/Home.php) (Kattge et al., 2020), BIEN database (http://bien.nceas.ucsb.edu/bien/) (Maitner et al., 2018) and published literature (e.g., Moles et al., 2009). We requested data of plant height (Trait ID 3106) from the database by registering and creating an account in July 2021. Data requested from TRY were released with a permission to free downloading 2 weeks after request. We used R BIEN to access the BIEN data. To obtain an average value of adult plant height of each species, the mean value of each species was calculated by merging all available data. Data on the pollination vectors for the species in our database were gathered from the TRY Plant Trait Database, published papers, and theses (Bawa et al., 1985; Sandor, 2012). Anemophilous plants refer to those pollinated exclusively by wind, while entomophilous plants refer to those pollinated by general insects, mainly including bees, bumble bees, butterflies, wasps, beetles, moths, and etc. We ensured that only animals associated with them were matched to each selected plant. However, in rare cases, plants pollinated by both anemophily and entomophily or by vertebrates were excluded from the dataset. We did not include life-history traits such as lifespan of plant species because of data unavailability. Information about life form (tree vs. non-tree) was added according to a look-up table of categorical plant-traits30 (https://www.try-db.org/TryWeb/Data.php#3). Overall, the final data with complete information (e.g., D, adult plant height, pollination vector, life form, and type of Spermatophytes) were restricted to the 158 plant species. All variables were prior to analysis log-transformed to correct for skewness in the trait distributions. A full list of species and data was provided in **Appendix S1** of the **Supplementary Material**.

### Statistical analysis

All analyses were performed in R (version 4.0, R Development Core Team, 2021). The phylogeny in mast seeding intensity, adult plant height, life form, pollination vector, and type of spermatophytes was constructed at the species level from a mega-tree (GBOTB.extended.tre) using the plant phylogeny for vascular plants (Jin and Qian, 2019). The name of the species in our dataset was checked using the Plant List database v.1.1 (http://www.theplantlist.org/) in R package Taxonstand (Cayuela and Oksanen, 2021).

We calculated Pagel’s λ to quantitively estimate if the similarity of mast seeding intensity and adult plant height between species is correlated with the phylogenetic similarity of plant species (Pagel, 1999). We used a randomization test by running the package ‘phytools’ (Revell, 2012) in R to test for the significance of λ. Pagel’s λ ranges from 0 to 1, with a λ of 0 indicating no phylogenetic signal and a λ of 1 indicating the strongest phylogenetic signal (Pagel, 1999).

The strength of the phylogenetic signal of binary traits, such as life form, pollination vector, and type of spermatophytes, was tested using the D statistic (Fritz and Purvis, 2010), implemented using the package ‘caper’ in R (Orme et al., 2013). If D is not significantly different from 0 (probability of Brownian phylogenetic structure, Pb > 0.05), the trait in question is supposed to evolve according to a Brownian evolution process. On the contrary, if the value of D is equal to or not significantly different from 1 (probability of random phylogenetic structure, Pr > 0.05), then a weak phylogenetic signal in trait is expected.

To investigate the association of mast seeding intensity with adult plant height, life form, pollination vectors as well as type of spermatophytes, we applied a multivariate phylogenetic generalized linear mixed model (PGLMM) to incorporate phylogenetic information and then correct for phylogenetic effects among species. We used a Gaussian distribution with phylogenetic trees, implemented in the R packages ‘phyr’ and ‘ape’ (Paradis and Schliep, 2019; Li et al., 2020). We considered mast seeding intensity as the response variable, adult plant height, life form, pollination vectors, and type of Spermatophytes as the predictor variables and phylogeny as a random intercept. The linear relationship between adult plant height and mast seeding intensity was evaluated by using univariate PGLMM at the species level rather than individual level, because of different origins of datasets. We explored the evolutionary link between adult plant height and mast seeding intensity using pairwise correlations that correct for phylogeny (Fernández-Martínez et al., 2019).

To distinguish the relative contributions of phylogeny, adult plant height, life form, pollination vectors, and type of Spermatophytes to the variation in mast seeding intensity across plant species, we used partial R^2^s for the logistic regression model (Ives, 2019) implemented by the R package “rr2” (Ives and Li, 2018). The partial R^2^_lik_ for each factor was calculated by comparing the full model with reduced models in which a given factor was removed and measuring the consequent reduction in likelihood (Wang et al., 2022).

## Results

In total, 158 seed plant species were analyzed in our study, belonging to 95 genera and 48 families. Among the plant species, 80 of them are pollinated by wind, while 78 are entomophilous species. Additionally, 123 species belong to angiosperm and 35 are gymnosperm.

### Phylogenetic signal in functional traits

**Figure 1:**
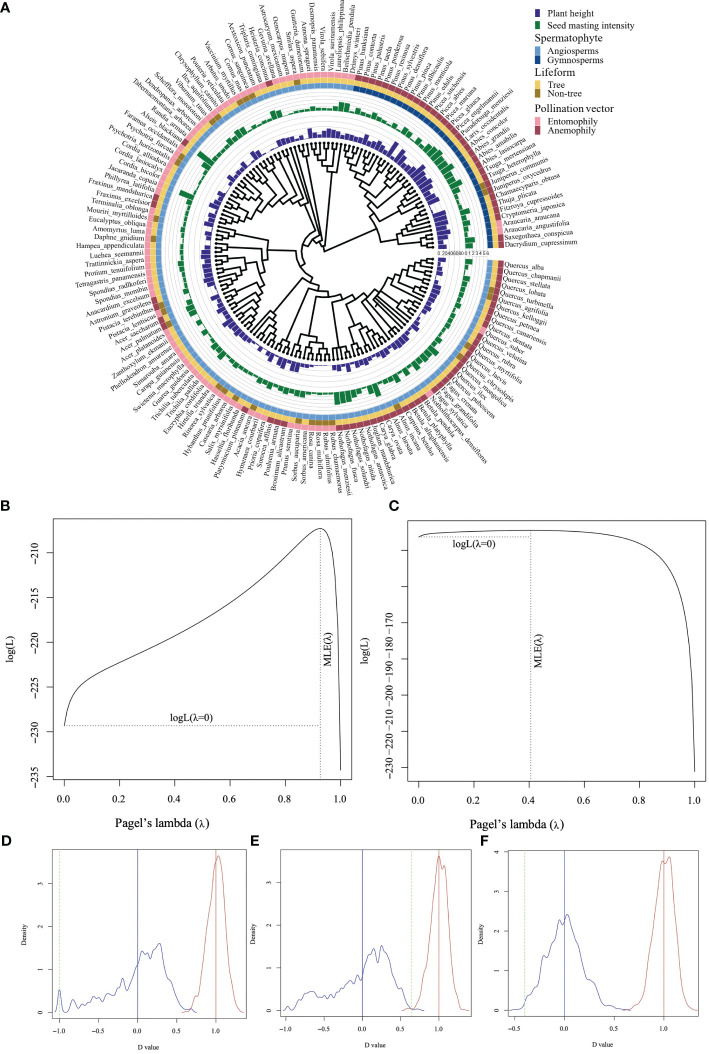
Plant height and mast seeding intensity D of Spermatophytes with different life form and pollination vectors mapped onto a plant phylogeny **(A)** and phylogenetic signal tests for plant height **(B)**, D **(C)**, type of Spermatophytes **(D)**, life form **(E)**, and pollination vector **(F)**. The green dashed line indicates the observed statistic D value, the blue distribution and solid line indicate a Brownian evolution motion and the red distribution and solid line indicate a random model. A marginally significant phylogenetic signal of D was detected based on the observed Pagel’s lambda **(C)**.

The phylogenetic signal of mast seeding intensity was marginally significant (Pagel’s λ = 0.406; P = 0.058). However, there was a strong phylogenetic signal of adult plant height (Pagel’s λ = 0.926; P< 0.001; **Figure 1**). The phylogenetic signal test showed that the type of Spermatophytes followed a Brownian model (D = -0.999, Pr = 0, Pb = 0.973). A strong phylogenetic signal was detected in the pollination vectors (D = -0.399, Pr = 0, Pb = 0.997) rather than in the life form (D = 0.641, Pr = 0.004, Pb = 0.003; **Figure 1**).

### Differences in adult plant height

**Figure 2:**
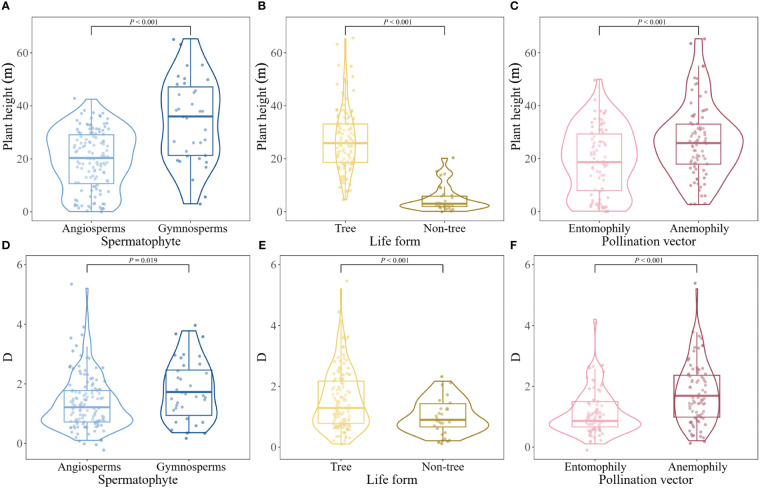
Difference in plant height **(A–C)** and mast seeding intensity **(D–F)** of Spermatophytes with different life form and pollination vectors.

There were significant differences in adult plant height between angiosperms and gymnosperms (t = -5.133, P< 0.001), trees and non-tree species (t = -15.87, P< 0.001), as well as anemophilous and entomophilous species (t = -3.566, P< 0.001; **Figures 2A–C**). Significant differences in mast seeding intensity were detected between angiosperms and gymnosperms (t = -2.373, P = 0.019), trees and non-tree species (t = -4.040, P< 0.001), anemophilous and entomophilous species (t = -4.879, P< 0.001; **Figures 2D–F**).

### Correlation of adult plant height to masting intensity

**Figure 3:**
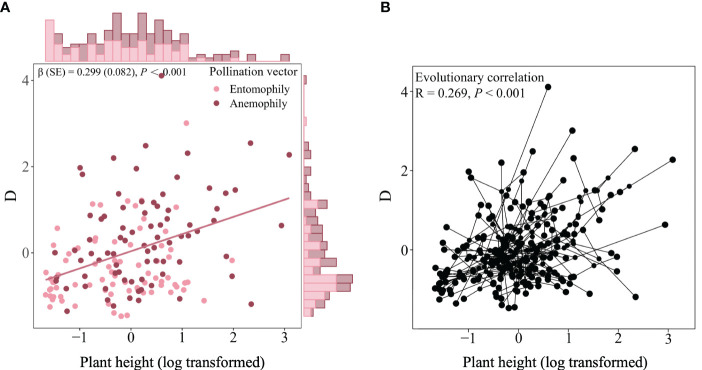
Linear correlation **(A)** and evolutionary link **(B)** between plant height and mast seeding intensity (D). β and SE stands for Estimate and standard error, respectively. Marginal histography indicates unimodal distributions of the log _10_-transformed plant height (x-axis) and log _10_-transformed mast seeding intensity (Y-axis).

The univariate PGLMM showed that there was a clear linear relationship between adult plant height and mast seeding intensity (z = 3.638, P< 0.001), which was likely attributed to the close correlation in anemophily rather than entomophily (z = 2.291, P = 0.022; z = 1.696, P = 0.090; **Figure 3**). Furthermore, adult plant height was evolutionarily correlated with and mast seeding intensity (R = 0.269, P< 0.001; **Figure 3**), indicating that taller plant species will exhibit stronger mast seeding intensity. The best-fitting multivariate PGLMM on mast seeding intensity also showed that adult plant height and pollination vectors appeared to be significant predictors (**Table 1**). However, life form and the type of Spermatophytes did not show significant effects on mast seeding intensity (**Table 1**).

**Table 1:** Multivariate PGLMM model constructed with mast seeding intensity (D) as response variable.

Predictor variable	AIC	Estimate (SE)	z	*P*
Intercept	428.8	-0.273 (0.221)	-1.233	0.218
Plant height		0.288 (0.101)	2.837	0.005
Life form		-0.010 (0.238)	-0.043	0.966
Spermatophyte		-0.225 (0.212)	-1.063	0.288
Pollination vector		0.654 (0.162)	4.040	< 0.001

**Figure 4:**
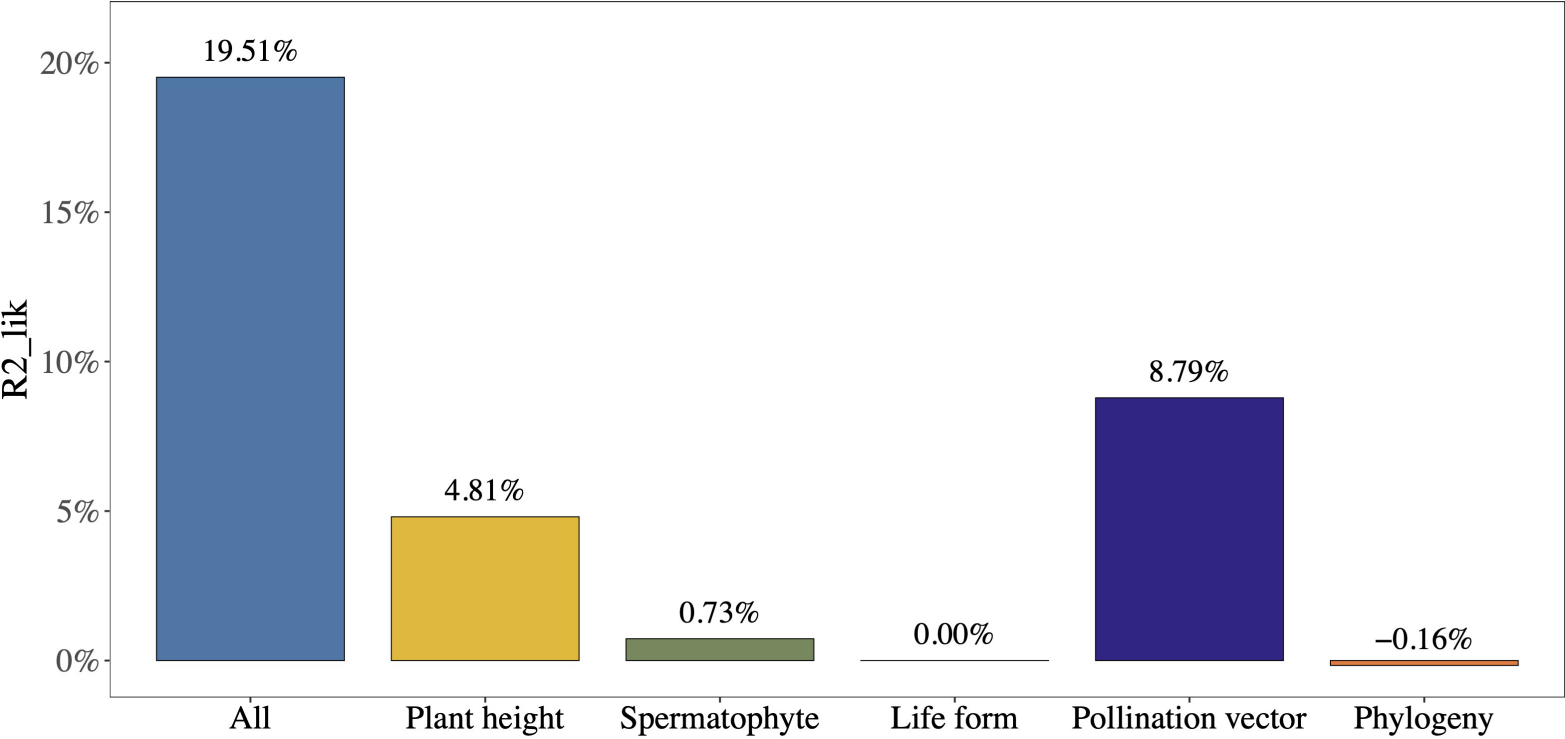
The relative contribution of different factors to the variations in mast seeding intensity (D) using partial R^2^ for the logistic regression model. Plant height and pollination vector explain a majority of variations in D.

Adult plant height and pollination vector explained the vast majority of the variation in mast seeding intensity between plant species (partial R^2^_lik_ = 4.81%, ΔlogLik = 3.89, P = 0.005; R^2^_lik_ = 8.79%, ΔlogLik = 7.27, P< 0.001; **Figure 4**), while phylogeny, life form, and type of Spermatophytes explained a minority of variation in mast seeding intensity (partial R^2^_lik_ = -0.16%, ΔlogLik = -0.130, P = 1.000; R^2^_lik_ = 0.00%, ΔlogLik = 0.0005, P = 0.97; R^2^_lik_ = 0.73%, ΔlogLik = 0.579, P = 0.28; **Figure 4**).

## Discussion

Our results provide evidence that mast seeding intensity is closely related to adult plant height than ever thought, supporting our first prediction stating that masting evolves predominantly in taller species. Our analyses also support our second hypothesis stating that mast seeding is stronger in anemophily than in entomophily that exhibits smaller plant height. Although mast seeding is related to phylogeny, life form, and type of Spermatophytes, they contribute little to variation of seed production of perennial plants.

We found that the phylogenetic signal of the mast seeding intensity is distributed unimodally like a Brownian model, implying that the common ancestry can potentially account for the high frequency of mast seeding. The phylogenetic signal observed in mast seeding intensity may suggest moderate phylogenetic conservatism for masting behavior, as observed by previous studies (Tanentzap and Monks, 2018; Pearse et al., 2020). The strong phylogenetic signal observed in adult plant height and the evolutionary correlation between mast seeding intensity and adult plant height convey the main findings in our study that mast seeding is more likely to evolve in tall plants, like gymnosperms, trees, and wind-pollinated species in our study.

Regardless of plant phylogeny, we found a strong positive association between mast seeding intensity and adult plant height, indicating that taller plants tend to have highly intermittent synchronous production of large seed crops. Economies of scale have been demonstrated to provide the ultimate factor driving the evolution of masting (Norton and Kelly, 1988; Koenig, 2021). How plant height selects for evolution of masting is of great importance to our deep understanding of masting from ecological and evolutionary perspectives. As EOS is the main selective force for masting (Kelly and Sork, 2002), the close correlation between adult plant height and mast seeding intensity implies that increased plant height may also provide EOS through either enhancing pollination efficiency and/or increasing satiation of seed predators. Strong correlations between plant height and flower number (Gomez, 2003; Trunschke et al., 2017), as well as increased attraction of pollinators, pollination success, and modularity of frugivorous network with plant height have been well observed (Klinkhamer et al., 1989; Weber and Kolb, 2013; Dupont et al., 2014; Paixão et al., 2023), which are important for enhancing pollination efficiency. Evidence has shown that increased plant height significantly promotes pollen dispersal and then strongly increases pollination success (Friedman and Barrett, 2009, 2011; Harder and Johnson, 2009; Sletvold et al., 2010; Aljiboury and Friedman, 2022), which also provides EOS. Additionally, plant height is positively correlated with seed dispersal distances (Burd and Allen, 1988; Thomson et al., 2011), which benefits from density-dependent seed survival via increased seed dispersal distances (Jansen et al., 2014), providing an alternative EOS. Moreover, plant height not only reduces herbivory but also has significant suppressive effects on pre-dispersal seed predation (Cariveau et al., 2004; Zhang et al., 2023), reflecting contributions of economies of scale in satiating predators. In addition, taller strategy of plant species incurs a number of costs, including disadvantaged water transport in vascular system, increased breakage risk, reduced investment in leaf biomass, and decreased leaf nitrogen content (Falster and Westoby, 2003). Consequently, the cost incurred by taller strategy of plant species is expected to serve as a potential selective pressure for mast seeding. Plant height is also strongly correlated with lifespan (Moles et al., 2009); therefore taller strategy of long-lived plants will benefit from mast seeding rather than regular seed production because they need to continuously allocate resources for the rest of their lives. Therefore, predominant appearance of masting behavior in taller plant species in our study would explain the higher masting intensity in gymnosperms, trees, and wind-pollinated species that widely adopt a taller strategy.

Our study clearly showed that mast seeding intensity of the anemophilous species is much higher than that of entomophilous ones, consistent with the previous studies predicting that wind-pollinated species had higher interannual variability of seed crops than animal-pollinated ones (Herrera et al., 1998; Kelly and Sork, 2002). These observations are possibly due to the fact that animal-pollinated species are usually at high risk to satiate the pollinators (Sork et al., 1993; Kelly et al., 2001; Xiao, 2022). The strong selective effect of the pollination vector (e.g., anemophily and entomophily) on mast seeding intensity indicates that, in the plant taxa, both pollination vector and plant height play important roles in the evolution of the mast seeding of plants. In our study, phylogeny, life form and type of Spermatophytes had much less power to explain mast seeding intensity than adult plant height and pollination vector, though they were also closely related to mast seeding intensity. This pattern can be partially explained by the overshadowing effects of adult plant height and pollination vectors. Our results also provide evidence that wind pollination exerts selection pressure on plant height of both gymnosperms and angiosperms, which can increase pollination efficiency and may also promote seed dispersal and survival rates of plants by overcoming pollen limitation or seed predation. Therefore, it seems that plant height provides direct selection pressure on mast seeding intensity of anemophilous and entomophilous plants, although the overall contributions of pollination vectors to the interannual variation in seed production are higher than plant height.

We admit that temperature, precipitation, soil fertility, seed predators, and plant genetic regulation may influence mast seeding. Moreover, plant height is a highly plastic trait within species as well as among species. However, our study using mean values, which may avoid the influence of these variants at individual level, clearly demonstrates that adult plant height is positively correlated with mast seeding intensity of 158 plant species in 95 genera and 48 families. For the first time, we demonstrate that adult plant height may act as a potential selective pressure for mast seeding, especially in wind-pollinated species. Pollination vectors appear to be critical in selecting plant height across Spermatophytes and have an indirect effect on mast seeding intensity of perennial plants. Given the selection force of pollination vectors on the evolution of plant height, the delicate correlations between plant height and mast seeding intensity provide a novel avenue for better understanding of the evolution of masting across plant species, which has important implications for the population dynamics, community structure as well as spatial distribution of plants and the associated fauna. This study will broaden our understanding of the evolution of masting by illustrating its relationship between functional traits of perennial plants, which highlights an ambitious direction for further research into other related traits (e.g., leaf area, photosynthetic capacity and transport of water and minerals in xylem) underlying variations in seed crops across plant species.

## Data availability statement

The datasets presented in this study can be found in online repositories. The names of the repository/repositories and accession number(s) can be found below: https://doi.org/10.6084/m9.figshare.22353517.

## Author contributions

HQ: Data curation, Formal analysis, Funding acquisition, Writing – original draft. XY: Data curation, Formal analysis, Writing – original draft, Writing – review & editing.
